# Idiopathic Sclerochoroidal Calcification With a Concurrent COL11A2 Variant: A Case Report

**DOI:** 10.7759/cureus.92271

**Published:** 2025-09-14

**Authors:** Ricardo A Murati Calderon, Victor M Villegas, Armando Oliver

**Affiliations:** 1 Ophthalmology, University of Puerto Rico, Medical Sciences Campus, San Juan, PRI

**Keywords:** choroidal calcification, choroidal lesion, coll11a2, collagen disorder, ocular imaging

## Abstract

Sclerochoroidal calcification (SCC) is a rare ophthalmic finding often discovered during routine evaluation, characterized by calcium deposition within the sclera and choroid. We report a patient with bilateral SCC and a concurrent heterozygous *COL11A2* variant. The patient reported gradual bilateral vision changes over several months. A fundus examination revealed yellow-white placoid lesions superior to the macula in both eyes, consistent with SCC. Multimodal imaging, including fundus autofluorescence (FAF), optical coherence tomography, and B-scan ultrasonography, confirmed the presence of calcified lesions with associated posterior shadowing. An initial metabolic and endocrine evaluation was unremarkable; however, genetic testing later revealed a heterozygous variant of uncertain significance in the *COL11A2* gene. Because *COL11A2 *mutations are typically linked to non-ocular Stickler syndrome (Type III) and no segregation or functional data are available, no causal inference or association with SCC can be made. We emphasize that this hypothesis is preliminary and based on a single co-occurrence. To our knowledge, reported cases of SCC with concurrent inherited collagen disorders are lacking. Documenting such co-occurrences might facilitate future case aggregation and help characterize the clinical spectrum of SCC. Consideration of heritable connective tissue disease in patients with idiopathic SCC, particularly when standard evaluations are inconclusive, may broaden our current understanding; however, further studies are needed to determine whether any meaningful relationship exists.

## Introduction

Sclerochoroidal calcification (SCC) was first described by Wong et al. as a rare ophthalmic finding characterized by calcium deposition within the sclera and involving the choroid [1]. The lesions are usually discovered during a routine ophthalmoscopic evaluation and appear as multiple yellow-white plaques, usually in the mid-peripheral fundus [2]. These lesions typically occur in older white individuals with no sex predilection [3]. Its incidence remains poorly defined due to its often asymptomatic and unrecognized presentation.

SCC can occur either idiopathically or in association with systemic metabolic disorders [4]. Reported systemic associations include disorders such as hyperparathyroidism, chondrocalcinosis, vitamin D intoxication, and chronic kidney disease [5]. Collagen disorders, particularly connective tissue diseases, can also be associated with SCC [6]. These disorders include autoimmune and heritable conditions that affect collagen-containing structures and that can lead to various ocular complications [7]. Collagen is a major structural component of the choroid and is essential to maintaining ocular integrity [8,9]. Collagen XI-related disorders, such as Stickler syndrome, have been implicated in various ophthalmic abnormalities, including myopia, vitreoretinal degeneration, and retinal detachment [10]. However, SCC has infrequently been reported as being associated with any of these conditions.

Distinct imaging techniques are essential for diagnosing and differentiating SCC from other choroidal pathologies. Fundus photography reveals the characteristic yellowish plaques, while optical coherence tomography (OCT) shows the elevations at the sclerochoroidal junction [3,5]. B-scan ultrasonography helps confirm the presence of calcification by showing hyperechoic lesions with posterior shadowing [4].

Given the rarity of SCC and its potential overlap with systemic disorders, further research is needed to elucidate its pathogenesis and clinical implications. Here, we present the case of an elderly man with SCC, contributing to the growing body of literature on its systemic associations, particularly in the context of collagen-related disorders.

## Case presentation

A 79-year-old Hispanic male patient with a history of arterial hypertension, atrial fibrillation, and type 2 diabetes mellitus presented with a gradual worsening of his already blurred bilateral vision over the past several months. Systemic evaluation for fever, chills, arthralgia, skin rash, dizziness, headache, recent travel, night sweats, and weight loss was insignificant. There was no notable family or ocular history. His social history was otherwise unremarkable.

Upon comprehensive ophthalmic evaluation, the patient’s best corrected visual acuity was 20/30 in both eyes (OU). Refraction measured +0.75 -2.25 x100 in the right eye (OD) and +0.75 -1.75 x80 in the left eye (OS). Intraocular pressures measured by applanation tonometry were 15 mmHg, OD, and 16 mmHg, OS. His pupils were equal, round, and reactive to light, with no relative afferent pupillary defect. Extraocular movements were full in all directions of gaze OU and without pain. Confrontation visual fields were full to finger counting in all quadrants OU.

The external examination was notable for a melanotic lesion on the right eyelid. A slit-lamp examination was unremarkable aside from 2+ nuclear sclerosis OU.

**Figure 1:**
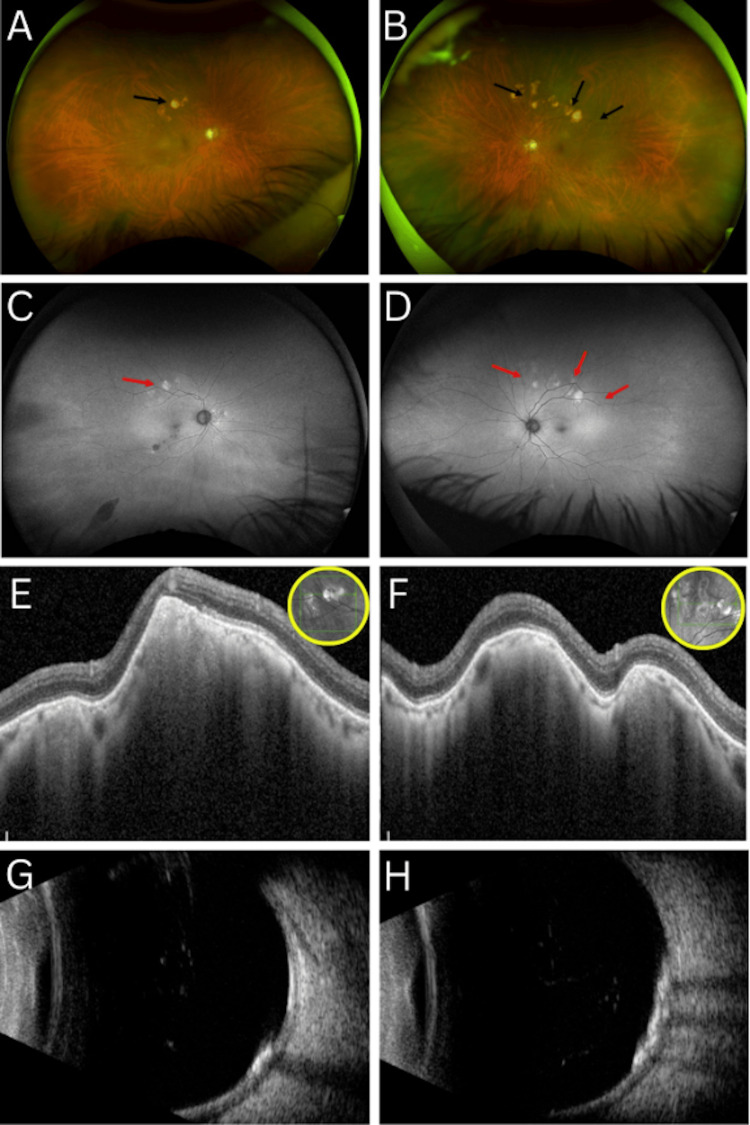
Multimodal imaging of both eyes at presentation Color fundus photographs at initial presentation of the right (A) and left (B) eyes show bilateral yellowish placoid lesions (black arrowheads) superior to the macula with associated drusen. FAF of the right (C) and left (D) eyes demonstrates mixed hyper- and hypoautofluorescent signals (red arrowheads) corresponding to the lesions. (E-F) OCT reveals choroidal elevations with increased reflectivity, while (G-H) B-scan ultrasonography confirms calcification with hyperechoic lesions and posterior acoustic shadowing in both eyes. FAF, fundus autofluorescence; OCT, optical coherence tomography

A dilated fundus examination of the OD (Figure 1A) showed optic disc cupping of 0.8, macular retinal drusen, and a large yellowish pigmented lesion superior to the macula, as well as congenital hypertrophy of the retinal pigment epithelium in the inferotemporal periphery. The OS (Figure 1B) revealed similar findings, with optic disc cupping of 0.8, macular retinal drusen, and multiple large yellowish pigmented lesions superior to the macula. Posterior vitreous detachments (PVDs) were present OU. Fundus autofluorescence (FAF) imaging of the OD and OS (Figure 1C, 1D) demonstrated multiple hypoautofluorescent lesions in the macular region of the OD, along with hyperautofluorescent signals corresponding to the yellow pigmented lesions located superior to the macula bilaterally.

High-resolution OCT scans of the OD and OS (Figure 1E, 1F) through the yellow lesions revealed hyperechoic choroidal lesions bilaterally. B-scan ultrasonography of the OD and OS (Figure 1G, 1H) revealed PVDs and multiple hyperechoic lesions in the posterior pole with associated acoustic shadowing OU.

A presumptive diagnosis of SCC was made following a referral to a retinal and ocular oncology specialist. A workup for calcium metabolic disorders was conducted, including a comprehensive metabolic panel and the measurement of bone-specific alkaline phosphatase (ALP), magnesium, and parathyroid hormone (PTH) levels. The laboratory results (Table 1) revealed a mildly elevated ALP of 131 and chloride levels of 108, while PTH and magnesium were within normal limits. Subsequently, the patient was referred to an endocrinologist for further assessment of a possible systemic disorder contributing to SCC.

**Table 1:** Laboratory workup ALP, alkaline phosphatase; BUN, blood urea nitrogen; eGFR, estimated glomerular filtration rate; PTH, parathyroid hormone; TSH, thyroid-stimulating hormone

Component	Result	Standard range
BUN	16 mg/dL	7-25 mg/dL
Creatinine	0.9 mg/dL	0.7-1.25 mg/dL
eGFR	87 mL/min/1.73 m²	>60 mL/min/1.73 m²
BUN/creatinine ratio	18	6-22
Calcium	9.3 mg/dL	8.6-10.3 mg/dL
ALP	131 U/L	38-126 U/L
PTH	37.1 pg/mL	14-65 pg/mL
TSH	1.88 mlU/L	0.5-5.0 mlU/L

Genetic testing was conducted using a whole blood sample, and a next-generation sequencing (NGS) diagnostic test (Invitae Inherited Retinal Disorders Panel; Invitae Corporation, San Francisco, CA, USA) was performed to evaluate over 330 genes associated with inherited retinal genetic diseases. NGS and deletion and duplication analysis done using the Invitae Panel showed a heterozygous mutation in the* COL11A2* gene. The variant was c.4541G>A (p.Arg1514His) (Table 2). This mutation was classified as a variant of uncertain significance (VUS). No syndromic features were identified on history or examination.

**Table 2:** Sequence variant

Gene and transcript	DNA variants, predicted effects, and zygosity	ClinVar ID	Highest allele frequency in a gnomAD population	In silico missense predictions	Interpretation
*COL11A2*, NM_080680.2	c.4541G>A, p.Arg1514His, Heterozygous	1305828	0.01%	Damaging	Uncertain significance

At the two-month follow-up, the patient’s fundus findings remained stable, as they had been in the initial evaluation, with no reported improvement in the visual symptoms.

## Discussion

SCC is an uncommon ophthalmic finding characterized by yellow-white placoid lesions typically located in the mid-peripheral fundus [4]. Shields et al. described SCC in 179 eyes, noting a predominance in older individuals, no strong sex predilection, and frequent incidental discovery during routine examination [11]. Our patient, a 79-year-old man, exhibited bilateral, mid-peripheral lesions consistent with the typical demographic and fundoscopic findings.

Although the etiology of SCC is idiopathic in many cases, systemic associations have been reported in the literature [2,5]. Shields et al. and others have linked SCC to calcium-phosphate imbalance and metabolic conditions such as primary hyperparathyroidism, chronic kidney disease, vitamin D intoxication, and renal tubular disorder (e.g., Bartter and Gitelman syndromes) [5,11]. Additional reports have associated SCC with CTDs and extracellular matrix dysregulation [12]. For example, Nabih et al. documented the occurrence of SCC in patients with chondrocalcinosis and tumoral calcinosis, both of which involve disordered calcium metabolism and extracellular matrix alterations, suggesting a pathogenic role for dysregulation of the extracellular matrix within the local stromal microenvironment [13]. Such dysregulation may reflect localized disturbances in extracellular matrix composition, calcification pathways, or stromal cellular signaling. In our case, the initial metabolic and endocrine evaluation was unremarkable. However, a more extensive systemic workup identified a genetically confirmed heterozygous *COL11A2 *VUS. Given the single-patient nature of this report and the absence of family or functional data, these results should be viewed cautiously as a co-occurrence rather than evidence of pathogenesis.

Multimodal imaging plays a key role in confirming the diagnosis of SCC and distinguishing it from other choroidal pathologies, including malignancies. On funduscopic examination, SCC typically presents as well-circumscribed, yellow-white placoid lesions in the mid-peripheral fundus, the superotemporal quadrant in particular, as described in the Shields et al. case series of 179 eyes with SCC [11]. Consistent with this description, our patient exhibited bilateral, mid-peripheral yellowish plaques, primarily located superior to the macula. Fortes et al. further demonstrated that FAF imaging of SCC lesions typically reveals a mix of hyper- and hypoautofluorescent signals corresponding to the calcified areas [14]. In our case, FAF imaging revealed both hypoautofluorescent macular changes in the OD and areas of hyperautofluorescence overlying the peripheral yellow plaques OU. While Shields et al. categorized SCC into four OCT morphologies, such as flat, rolling, rocky, and table mountain (each often associated with choroidal thinning and outer retinal changes), our patient’s macular OCT was unremarkable, likely due to the peripheral location of the calcified lesions [11]. Finally, B-scan ultrasonography, a critical modality for identifying calcification, demonstrated hyperechoic lesions with posterior acoustic shadowing OU, a hallmark feature of SCC. These imaging findings align with those reported in the literature and support the use of comprehensive multimodal imaging for the accurate diagnosis and exclusion of more worrisome choroidal entities.

Our case is notable for the presence of bilateral SCC in a patient with a concurrent heterozygous VUS in the *COL11A2 *gene. Usually, *COL2A1 *and *COL11A1 *mutations are associated with ocular Stickler syndrome, which is marked by high myopia, vitreoretinal degeneration, and retinal detachment. However, *COL11A2 *mutations are typically linked to the non-ocular, autosomal recessive form (Type III) [10,15]. Sirko-Osadsa et al. described a multigenerational family with *COL11A2 *mutations presenting with craniofacial features and hearing loss, but no ocular involvement [15]. In this context, we report the *COL11A2 *variant in our patient as a concurrent finding; in the absence of segregation or functional data, no causal inference or association with SCC can be made. While extracellular-matrix dysregulation remains a plausible mechanistic pathway for calcification in general, this single-patient observation is hypothesis-generated only. Nevertheless, documenting such co-occurrences, together with standardized imaging and laboratory details, may help expand the phenotypic catalog for *COL11A2* and support future aggregation, segregation, and functional studies.

This case underscores the importance of considering genetic testing as an adjunct, particularly when initial metabolic workups are unremarkable in patients with idiopathic SCC. However, positive results, particularly for VUS, should be interpreted cautiously and not presumed to underlie SCC. To our knowledge, this is the first reported co-occurrence of SCC in a patient with a genetically confirmed *COL11A2 *VUS. Limitations of this report include its single-patient design and the lack of family segregation studies, as the patient comes from a poor social support system with no family members available to participate. This case highlights the value of a multidisciplinary approach, including endocrinologic and genetic evaluation, when metabolic causes are not identified. Further research is needed to clarify the molecular pathways linking extracellular matrix dysregulation to SCC and to explore whether specific collagen-gene variants may predispose individuals to this uncommon ocular finding of SCC.

## Conclusions

This case describes bilateral SCC in the presence of a concurrent heterozygous *COL11A2* VUS, with no clear metabolic or systemic etiology. While most SCCs are considered idiopathic or secondary to metabolic disturbances, our observations raise the possibility that extracellular matrix abnormalities might play a role. However, as this is a single case with no family or functional data, no causal inference or association can be made from this single observation; at most, it is hypothesis-generating. When routine metabolic testing is unremarkable, genetic testing might provide useful information, but findings, especially VUS, should be interpreted with caution. Our study is limited by single-patient data and a lack of family segregation information.

Given the expanding role of genetics in ophthalmic diagnostics, our report highlights the potential utility of genetic testing in patients with unexplained SCC while emphasizing that VUS findings should be interpreted with caution. Most SCC cases are considered idiopathic or secondary to systemic metabolic dysregulation, which was not present in our case. The identification of a concurrent *COL11A2* variant in our patient represents a co-occurrence that, at present, should not be interpreted as evidence of a causal or associative relationship. Instead, this observation underscores the need for systematic reporting and follow-up studies to further characterize the spectrum of findings in SCC. Future studies incorporating larger case series, family segregation analyses, and genetic screening will be necessary to clarify whether any consistent patterns emerge regarding* COL11A2* or other collagen gene variants in SCC.
